# Uniting the un-united: should established non-unions of femoral shaft fractures initially treated with IM nails be treated by plate augmentation instead of exchange IM nailing? A systematic review

**DOI:** 10.1007/s11751-018-0323-0

**Published:** 2018-11-13

**Authors:** Gareth Medlock, Iain M. Stevenson, Alan J. Johnstone

**Affiliations:** Trauma and Orthopaedic Unit, Aberdeen Royal Infirmary, Wards 212/213, Foresterhill, Aberdeen, AB25 2ZN UK

**Keywords:** Femoral non-union, Femoral fracture, Intramedullary nailing, Adjunctive plating

## Abstract

The majority of femoral fractures are surgically treated with intramedullary nails. Non-union rate is low but challenging and costly if it occurs. There have been encouraging results from the use of augmentative plating as a treatment for non-union of femoral fractures. We performed a systematic review of the literature to compare union rates, time to union and complications between exchange nailing and augmentative plating as a primary procedure following a diagnosis of femoral non-union following initial nailing. We found a total of 21 papers, which found the mean union rate of augmentative plating to be 99.8% compared to 74% (*P* = 2.05^−12^) found for exchange nailing. Times to union were comparable at 5.9 months for augmentative plating and 6.3 months for exchange nailing (*P* = 0.68916), and complication rate was 4% for augmentative plating compared to 20% for exchange nailing. From the evidence available, plate augmentation provides a more reliable union rate if used as the first operative intervention on a non-union of a femoral fracture compared to exchange nailing.

*Level of Evidence IV* Systematic review of therapeutic studies.

## Background

In the western world, the majority of displaced femoral diaphyseal fractures are treated operatively using intramedullary nails (IMNs) with early return to function and a low incidence of complications [1]. When femoral non-unions do occur, the treatment options can be time consuming, challenging and expensive. Based upon a health economics study from the UK, the cost of treating a femoral non-union is at least £17,000 (US $22,000) per patient [2]. From the patients’ perspective, femoral non-unions result in on-going pain, altered gait, delayed return to work and psychosocial impairment. Therefore, treatment methods that improve the likelihood of fracture union combined with a reduced “time-to-union” are welcomed by clinicians and patients alike. Currently the most common method of treating femoral diaphyseal non-unions is to perform an exchange-nailing procedure, whereby the original IM nail is removed, and the femoral canal is reamed to stimulate the natural healing response. Reaming permits a larger diameter IMN to be inserted thus improving the mechanical stability. Despite this exchange, IM nailing is not as uniformly successful and persistent non-unions do occur.

An alternative treatment is the augmentation of the IM nail with a plate and screws, and this shows real promise at reducing the incidence of persistent non-union following IM nailing. If the clinical assessment determines the IMN has maintained its structural integrity and unlikely to fail before the fracture has healed, plate augmentation in isolation can be undertaken. One major advantage to this technique is that surgical exposure of the fracture site will permit the surgeon to remove fibrous tissue and freshen the fracture ends as a stimulus for healing; there is opportunity also for direct bone grafting at the surgeon’s discretion.

In this article, we have conducted a comprehensive systematic review of the published literature on exchange IM nailing and on plate augmentation of femoral diaphyseal fractures that were treated initially, but unsuccessfully, with IMNs.

## Methods

### Identification and eligibility of relevant studies

A comprehensive literature search using Medline, EMBase, Cochrane and CINAHL was conducted on the 11 April 2016, from inception to this date, identifying relevant studies using the key words and terms “femoral fracture non-union,” “exchange nailing” and “plate augmentation”. Inclusion criteria were papers written in English and limited to surgical procedures undertaken for aseptic femoral diaphyseal non-unions that had been treated with an IMN initially and with only one surgical revision procedure; infected non-unions were excluded. Other inclusion criteria were use of a measure of “time to union” by the same radiological method: this was bridging callus present on at least 3 cortices on the anteroposterior and lateral radiographs of the femur. Details of complications following surgery to treat the non-union were noted.

### Data extraction

Two reviewers screened the titles and the abstracts of all potentially relevant publications independently. Full articles were critiqued, and those judged to be eligible for consideration in the study were examined in greater detail. Any controversial papers were discussed in detail between the two reviewers. If doubt still existed regarding suitability for inclusion, the final decision for inclusion or exclusion was undertaken by the senior clinician. Each paper was then reviewed in turn to extract the following data using a standardised proforma including the following headings: study design, patient age, gender, treatment method used to treat the established femoral diaphyseal non-union, percentage of patients obtaining union after the index procedure and time to union according to established radiological criteria. A proportion of the papers had included patients who had had several previous operative attempts for established non-union prior to the index intervention. Only those papers that could provide a sufficient breakdown of each patient’s treatment history undertaken prior to the index procedure were included in our analysis. Only those individuals who had undergone one procedure after developing a non-union were considered. In addition to exchange nailing, some authors described combining this technique with open bone grafting of the fracture site for some patients within their series.

### Outcome measures

The primary outcome measure was radiographic union after the index intervention. Radiographic union was defined as bridging callus across at least three cortices on anteroposterior and lateral views of the femur. The secondary outcome measure was the time to union measured in months. The tertiary outcome measure was the incidence and severity of perioperative and postoperative complications. The prevalence of use of bone grafting in both techniques was recorded.

Due to significant study heterogeneity, particularly with regard to the methods and timing of patient follow-up following the index procedure, it was deemed inappropriate to pool the data from the published articles for meta-analysis. A narrative approach was used.

### Statistics

The Chi-squared test was used to compare the union rate between the two surgical treatments exchange nailing and augmentation plating. Time to union was compared using the Mann–Whitney U test between the two surgical interventions. Analysis was performed using the SPSS statistics software (SPSS Science Inc, Chicago, Illinios, USA).

## Results

**Fig. 1:**
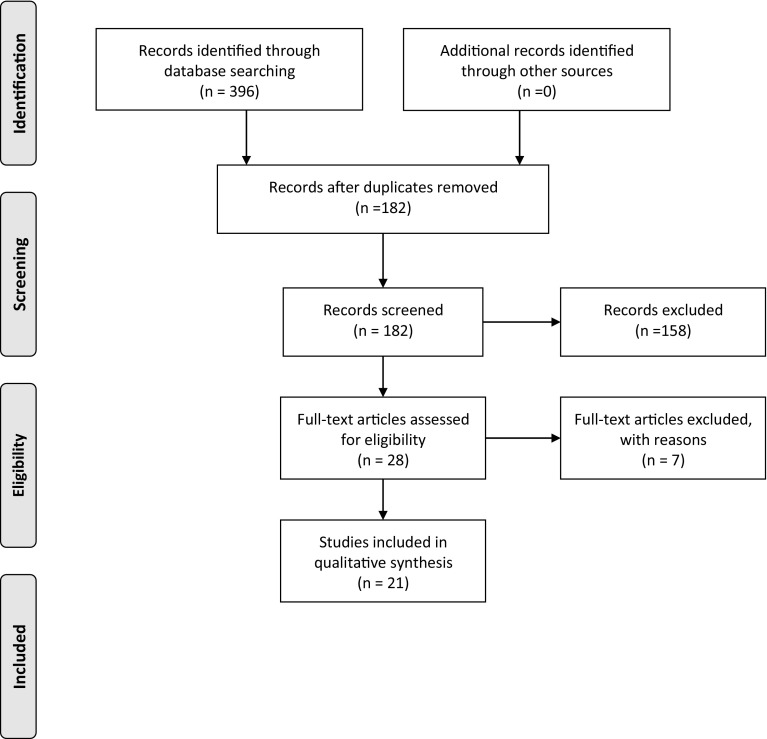
PRISMA method of systematic review. Moher et al. (2009) [31]

Our initial literature search identified 396 suitable studies for further evaluation. Removal of duplicated titles left 182 studies. Of these, 158 were omitted because the title or abstract did not fulfil the inclusion criteria. The remaining 28 studies were analysed in detail. Seven further studies were excluded subsequently because of either: (a) an inability to isolate patients that had undergone only one procedure to treat the non-union or; (b) where the time to radiographic union was not accurately recorded. The flowchart in Fig. 1 illustrates our compliance to the PRISMA method of systematic review. Twenty-one papers remained for analysis. The papers included: one cohort study comparing the two methods [3]; one randomised control trial comparing closed versus open bone-grafting techniques along with exchange nailing [4]; one cohort series comparing the effect of reaming size when exchanging the nail [5]; seven exchange-nailing case series [6–13]; one cohort series comparing augmented plating versus exchange plating [14]; and ten plate augmentation case series [15–23]. Tables 1 and 2 include details of the papers grouped according to exchange IMN and plate augmentation, respectively.

**Table 1:** Patients and results treated with exchange nailing

Year of publication	Author	Type of study	Definition of non-union	Number of femoral non-unions	Open fracture	Average age	Months until exchange nailing	Bone graft used	Months until union	*Radiographic union after first procedure*	*Complications*
2003	Banaszkiewicz P. A.	Case series	–	19	4	36	–	0	9	37	2 infected non-unions 2 broken nails
1999	Furlong A. J.	Case series	Pain on mobilisation Minimum of 6 months no progression on monthly radiographs for 3 months	25	2	39	–	12	7	96	Nil
2010	Park J.	Cohort series: Exchange nailing vs. plate augmentation	Pain on weight bearing Minimum of 6 months no progression on monthly radiographs for 3 months	5	–	40	15	0	6	20	Nil
2009	Shroeder J. E.	Case series	No radiographic union after 6 months and pain on weight bearing	35	6	32	–	0	4	94	Nil
2015	Swanson E. A.	Case series	Pain and no radiographic (plain radiograph or CT) improvement	50	8	40	–	0	6	72	Nil
2015	Tsang S. T.	Case series	–	20	–	38	8	3	9	85	Nil
2000	Weresh M. J.	Case series	Pain on weight bearing Minimum of 6 months no progression on monthly radiographs for 3 months	19	0	39	–	0	8	53	Nil
1997	Wu C.	Randomised controlled trial: closed vs. open bone-grafting techniques	–	18	–	37	–	–	5	100	Nil
2007	Wu C.	Cohort series: effect of reaming size	–	41	–	34	–	0	4	92	Nil
2002	Yu C. W.	Case series	No radiographic union after 1 year after treatment or another method is required to obtain union	25	0	38	20	0	6	92	Nil
				257		37	13		6.3	74

**Table 2:** Patients and results treated with augmentative plating

Year of publication	Author	Type of study	Definition of non-union	Number of femoral non-unions	Open fracture	Average age	Months until augmentative plating	Bone graft	Months until union	*Radiographic union after first procedure*	*Complications*
2010	Chen C. M.	Case series	–	50	–	44	–	50	6	100	1 superficial infection
2005	Choi Y. S.	Case series	Pain on weight bearing Minimum of 6 months no progression on monthly radiographs for 3 months	8	4	37	10	8	8	100	Nil
2011	Gao K. D.	Case series	Clinical and radiographic unhealed fracture	9	–	38	17	9	7	100	Nil
2011	Hakeos W. M.	Case series	Pain on weight bearing Minimum of 6 months no progression on monthly radiographs for 3 months	7	2	43	9	2 local 5 posterior iliac spine	6	100	1 superficial infection
2015	Jhunjhunwala H. R.	Case series	No evidence of radiographic union for greater than 6 months	40	1	35	6	24	4	98	1 infected non-union
2014	Jiang L.	Cohort series: Augmentation vs. exchange plating	Pain and no evidence of radiographic union greater than 6 months	12	–	46	12	12 anterior iliac spine	4	100	1 plate removal
2012	Lin C. J.	Case series	–	22	0	34	20	13 anterior iliac spine	5.5	100	Nil
2008	Nadkarni B.	Case series	Pain and no evidence of radiographic union	2	0	44	29	2 anterior iliac spine	6.5	100	Nil
2010	Park J.	Cohort series: Exchange nailing vs. plate augmentation	Pain on weight bearing Minimum of 6 months no progression on monthly radiographs for 3 months	4	–	48	11	4	8	100	Nil
2011	Said G. Z.	Case series	–	14	–	42	8	9 anterior iliac spine	4	100	1 superficial wound infection
1997	Ueng S. W.	Case series	Pain and no evidence of radiographic union	17	0	34	15	7	7	100	Nil
2012	Ye J.	Case series		6	0	47	12	6 anterior iliac spine	4.5	100	1 plate removal
				191		41	13.5		5.9	99.8	

The union rate with plate augmentation was 99.8% (190/191) compared to 74% (190/257) with exchange nailing. This difference was statistically significant (Chi-squared, *p* = 2.05^−12^). Time to radiographic union averaged 5.9 months (4–8 months) following plate augmentation in comparison with 6.3 months (4–9 months) following exchange nailing. No statistically significant difference was demonstrated using the Mann–Whitney U test (*p* = 0.68916).

Complications included persistence of the aseptic non-unions requiring yet further surgery and three deep infections at the site of non-union following exchange nailing. One deep infection occurred at the site of a non-union after plate augmentation. Plate augmentation was also associated with its own specific complications which included three superficial infections treated successfully with dressings and antibiotics and plate prominence requiring removal after union.

Bone grafting in the exchange-nailing cohort was used 10% of the time compared to 79% of the time in the augmented plate group. Unfortunately the data for these cases were not presented separately to enable analysis, but within the exchange-nailing group, studies that used bone grafting [5, 7, 10, 13] led to a union rate of 91%.

## Discussion

The current gold standard treatment for an established femoral diaphyseal non-union is exchange IM nailing. Our findings in a systematic review of the literature suggest there is evidence to support plate augmentation as an alternative approach. Direct comparisons in the literature between the two methods of treatment are difficult given the differences in study designs. However, there is a very high radiographic union rate achieved in all of the plate augmentation studies compared with the lower union rates of exchange nailing documented in 10 of the 11 studies included in our analysis. Where union was successful, the time to union was similar for both surgical techniques.

Most aseptic diaphyseal non-unions of the femur are thought to occur due to mechanical instability, predominantly rotational instability [24, 25]. Exchange nailing to a larger diameter nail goes some way to improving the biomechanical environment as larger diameter IMNs are torsionally stiffer. In addition, provided the intramedullary canal is not reamed excessively, better frictional contact can be achieved between the nail and surrounding bone by increasing the surface diameter of the IMN. Park et al. [26] share this opinion and have added that non-isthmal fractures decrease fracture stability since the IMN can no longer make contact with the inner aspect of the cortical bone at the level of the fracture. They postulate that fractures that occur outside the isthmus are prone to non-union since IMN-bone stability becomes increasingly dependent upon the stability offered by the proximal and distal locking screws that have significantly poorer biomechanical properties compared with the IMN itself. Another major consideration is that the majority of modern IMNs are made from titanium that have a Young’s modulus of approximately 60% of stainless steel. This alloy is the material of choice for many as it is more resistant to fatigue failure as compared with stainless steel. For this reason, the implant industry has manufactured IMNs that have very distally placed cross-screw holes without an associated high risk of IMN failure. Although these extreme distal locking options are advantageous for treating very distally placed fractures within the metaphyseal region of the femur, extreme distal locking adds no advantage when treating diaphyseal fractures. Long cross-screws are mechanically inferior to shorter ones and the degree of instability is further accentuated from the poorer hold that distally placed cross-screws have in the surrounding metaphyseal bone where the cortices are much thinner compared to the meta-diaphysis area. Titanium cross-screws are also more flexible than stainless steel screws, so the combination of increased cross-screw length and increased cross-screw flexibility can significantly reduce IMN-bone construct stability especially with respect to rotational forces [26]. Very distal locking options have perhaps led to an increased risk of non-union, and most of the papers assessed used these IMNs. Although it is recognised that IMNs are good at resisting bending forces and maintaining axial stability, their reduced rotational stability cannot be improved unless shorter and broader IM nails are used. These would permit shorter and, therefore, more mechanically stable cross-screws to be inserted. Careful consideration of the length of IMN that is required to treat each fracture type is needed without reverting to the long accepted “reinforcing the whole of the bone” concept. In this way, it may be possible to reduce the incidence of non-unions happening in the first instance and to improve the overall success rate after exchange nailing. However, plate augmentation would appear to be a promising alternative way of achieving fracture stability but avoiding the potentially negative effects to the abductor mechanism and soft tissues when exchanging an IMN.

The importance of rotational instability in femoral diaphyseal non-unions is exemplified by attempts to encourage union through dynamising femoral nails; the subsequent union rates are less than 50% and are associated with a risk of significant limb shortening by more than 2 cm [27–29]. Dynamisation increases axial loading of the fracture and should, in theory, encourage bone healing, but unless the bone ends can interlock in a way that restores rotational control, the instability and mechanical environment for healing will be made worse by the removal of one or more of the distal cross-screws.

Plate osteosynthesis used in isolation after removal of an intramedullary nail has been described in association with insertion of bone graft [30], but plates used alone are unpopular as patients have to comply with restricted weight bearing to reduce the risk of plate failure. By comparison, plate augmentation of a femur already stabilised by an IMN offers the advantages of a more favourable mechanical environment for bone healing and that of immediate weight bearing after surgery. In most of the papers reviewed, the preferred plate and screw system was a 4.5-mm compression plate, being either low contact or standard plate design. The recorded success suggests these implants are stiff enough to resist the rotational forces present at a femoral non-union and, when applied in a compression mode, may also help to limit excessive axial displacement.

Bone grafting was used more widely in the plating group as compared to the exchange-nailing group most likely because the non-union site was exposed during this procedure. Unfortunately, these patients’ data were never separated or analysed except for one paper [7] where a small improvement in union time was found but not statistically significant.

The complication rates for exchange nailing were 20% compared to 4% for plate augmentation. However, if failure to achieve satisfactory bone union is excluded, plate augmentation is associated with a 4% complication compared with 1% for exchange nailing. This may relate to the risk of infection that accompanies larger surgical approaches that communicate with the site of non-union. In comparison, revision nailing is not a benign procedure; often removal of the nail and insertion of another can lead to substantial damage to the abductor muscles and their insertions.

The limitations to this study include it being a systematic review of low-level evidence studies, mostly level IV case series, involving relatively small numbers of patients. The fractures are heterogenous. The definition of non-union differed in the papers which may have led to some over- or under-treatment of the non-union. Using radiographic time to union is notoriously difficult as opinions vary and the timing of clinical reviews is not consistent. The majority of the papers failed to perform patient-reported outcome measures.

## Conclusion

The results of plate augmentation are at least as good as and perhaps better than exchange IM nailing for treating established femoral diaphyseal non-unions that had been primarily stabilised using an IMN. The time to union following the plate augmentation or exchange nailing is similar.

Bone grafting around the fracture site can be an adjunct for successful union.

Both techniques are associated with a low incidence of complications. Although postoperative infection is more common following plate augmentation, the incidence of serious deep-seated infection would appear to be no higher when compared with exchange IMN.
